# Assessment of the interval between diagnosis and definitive treatment of lung cancer at a public institution in São Paulo

**DOI:** 10.1590/0100-6991e-20253836-en

**Published:** 2025-08-18

**Authors:** OTAVIO ROCHA FIUZA, BARBARA SIQUEIRA SOUTO MAIOR, LUÍSA BEISMAN DE MORAES, DANIEL AUGUSTO XAVIER CARVALHO, JOÃO ALÉSSIO JULIANO PERFEITO, ERNESTO EVANGELISTA, ANDRÉ MIOTTO

**Affiliations:** 1 - Escola Paulista de Medicina/Universidade Federal de São Paulo, Disciplina de Cirurgia Torácica - São Paulo - SP - Brasil

**Keywords:** Lung Neoplasms, Time-to-treatment, Thoracic Surgery, Diagnosis, Neoplasias Pulmonares, Tempo para o Tratamento, Cirurgia Torácica, Diagnóstico

## Abstract

**Introduction:**

This study aimed to investigate the interval between the diagnosis and definitive surgical treatment of lung cancer patients at a public institution in São Paulo.

**Method:**

A retrospective observational study was conducted, using medical records to collect data on the periods between the first abnormal chest computed tomography (CT) scan, the initial consultation with the specialist, and the subsequent tumor resection.

**Results:**

The analysis of 20 patients revealed a substantial average waiting period of 425.6 days between diagnosis and definitive treatment. During this interval, an average of 282 days elapsed between diagnosis and the initial specialist consultation, while the period between the first consultation and treatment averaged 143 days. By comparing the initial and final staging, 70% of the patients progressed to a higher stage over this period.

**Conclusions:**

The identified interval is concerning and exposes patients to elevated risks during this waiting period. This prolonged duration poses potential threats to patient health, resulting in decreased quality of life, increased risk of disease progression, reduced chances of cure, and diminished overall survival prospects. Addressing and minimizing this extended interval is crucial for improving patient outcomes and enhancing the effectiveness of lung cancer treatment.

## RESUMO

**Objetivo:**

Este estudo teve como objetivo investigar o intervalo entre o diagnóstico e o tratamento cirúrgico definitivo de pacientes com câncer de pulmão em uma instituição pública em São Paulo.

**Método:**

Foi conduzido um estudo observacional retrospectivo, utilizando registros médicos para coletar dados sobre os períodos entre a primeira tomografia computadorizada de tórax alterada, a consulta inicial com o médico especialista e a ressecção tumoral subsequente.

**Resultados:**

A análise de 20 pacientes revelou um período de espera médio de 425,6 dias entre o diagnóstico e o tratamento definitivo. Neste intervalo, uma média de 282 dias transcorreu entre o diagnóstico e a consulta inicial com o especialista, enquanto o período entre a primeira consulta e o tratamento teve uma média de 143 dias. Ao comparar os estadiamentos inicial e final, 70% dos pacientes progrediram de estágio ao longo desse período.

**Conclusões:**

O intervalo identificado é alarmante e expõe os pacientes a riscos elevados durante este período de espera. Essa duração prolongada representa potenciais ameaças à saúde dos pacientes, resultando em comprometimento da qualidade de vida, aumento do risco de progressão da doença, redução das chances de cura e diminuição das perspectivas gerais de sobrevida. Abordar e minimizar este intervalo prolongado é crucial para melhorar os resultados dos pacientes e aumentar a eficácia do tratamento do câncer de pulmão.

## INTRODUCTION

Lung cancer, a highly prevalent neoplasm, can be categorized into two main groups, small cell lung cancer (SCLC) and non-small cell lung cancer (NSCLC), the latter accounting for a substantial majority, constituting more than 87% of lung neoplasms^1^. In addition to its histological classification, discerning the specific type of lung cancer is crucial, as it directly influences the personalized treatment regimen to be instituted^1^ ^,^ ^2^. 

The insidious nature of this disease is responsible for concealing its presence, as its symptomatic manifestation remains elusive or absent until advanced stages, in which the tumor extends beyond the lung tissue^1^. This delay in symptomatic manifestation contributes to a high frequency of diagnosis at an advanced disease stage, requiring systemic therapeutic approaches, which increases treatment costs and compromises overall survival results^3^.

NSCLC has a variable tumor doubling time, which can occur between 20 and 300 days, although it is characterized by a faster mean doubling time, representing the heterogeneity and aggressive nature of this disease^4^. In addition, one study identified a correlation between the doubling time of lung neoplasms and their prognostic implications, revealing that tumors with a shorter doubling time tend to have more unfavorable prognoses compared with the ones with a longer doubling time^5^. One established the role of the disease evolution time in the prognosis of patients with lung cancer, it is evident that delays in treatment initiation allow tumor growth and local and distant dissemination, resulting in compromised survival rates and high risks of disease recurrence^6^ ^-^ ^8^.

Given the importance of timely treatment initiation for the outcomes of patients with NSCLC, this study aims to examine the interval between initial imaging diagnosis and subsequent surgical intervention for patients with lung cancer. By identifying the time intervals elapsed until treatment start for these patients, this research seeks to understand the related factors to enable therapeutic schedule optimization and, consequently, improve overall prognosis and survival outcomes for individuals with NSCLC.

## METHODS

This study was approved by the Ethics Committee of the Federal University of São Paulo (CAAE: 57974022.0.0000.5505, Opinion: 5.653.348).).

### Study design and setting

We conducted a retrospective, analytical, observational, and longitudinal cohort study at Hospital São Paulo, through the Thoracic Surgery Division of the Federal University of São Paulo/Paulista School of Medicine, a medical institution located in the city of São Paulo, Brazil

### Patients

The selection of participants included patients aged between 18 and 75 years, diagnosed with pulmonary malignant neoplasms, who underwent surgical resection of the tumor at Hospital São Paulo during the years 2021 and 2022. Exclusion criteria were individuals with advanced neoplasms who were not eligible for surgical treatment, those who chose not to undergo surgical treatment, and those who died within a period of less than 30 days.

### Data

The database was compiled by examining the electronic medical records of patients treated by the Thoracic Surgery Division at Hospital São Paulo, with the explicit consent of the participants. The dataset variables included sex, age, smoking history, immediate post-treatment outcomes, histological type of the tumor, disease stage, surgical technique employed for tumor resection, date of the first altered chest CT scan, date of the first consultation with the thoracic surgeon, and date of tumor surgical excision.

**Figure 1:**
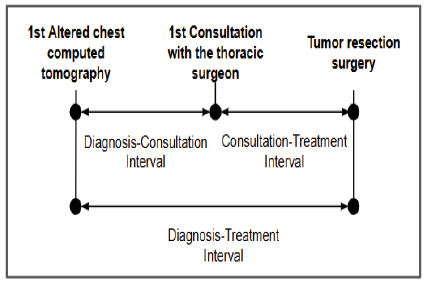
Schematic representation of the established intervals.

The date of the first altered chest CT scan was the milestone for the date of diagnosis. Subsequently, the intervals between diagnosis and consultation (Diagnosis-Consultation), between consultation and treatment (Consultation-Treatment), and between diagnosis and treatment (Diagnosis-Treatment) were established, as illustrated in Figure 1.

### Data analysis

For the statistical analysis, we used the Microsoft© Excel version 365 software, developed by Microsoft© Corp., located in Redmond, WA, USA. This software was used as an analytical tool to process data. The quantitative values obtained in the study were organized and described as mean, median, standard deviation, as well as minimum and maximum values, providing an exploration of central tendency and dispersion. In the case of qualitative data, the organization and description were conducted focusing on absolute and relative frequencies.

To identify significant patterns and relationships, we used the following statistical tests. The ANOVA test compared quantitative variables, while the Student’s t-test analyzed specific quantitative aspects. For qualitative variables and to determine the frequency of events, we used the Chi-square test. In all analyses, the significance level was set at 0.05 to control for Type 1 error.

## RESULTS

### Sample characteristics

As detailed in the methods section, this study selected a population composed of individuals who underwent lung resection surgery for the treatment of malignant lung neoplasms at Hospital São Paulo during the years 2021 and 2022. These individuals met the inclusion and exclusion criteria outlined in the previously mentioned section. A total of 20 participants contributed to the study, and none were excluded from the analysis. A detailed understanding of the characteristics of these participants can be identified in Table 1.

**Table 1:** Clinical and epidemiological profile of patients undergoing pulmonary resection surgery.

Variable	N	%
Age (years)	61,6	± 9.3
Variable	N	%
Sex		
Male	7	35
Female	13	65
Smoking/Ex-smoking		
≥20 pack-year	8	40
Histology		
Adenocarcinoma	8	40
Squamous cell carcinoma	5	25
Other	7	35
Initial stage		
I	17	85
II	2	10
III	1	5
Final stage		
I	11	65
II	4	10
III	5	25
Variable	N	%
Resections		
Segmentectomy	1	5
Lobectomy	17	85
Pneumonectomy	2	10

The age distribution in our cohort ranged from 40 to 75 years, reflecting a diverse demographic. The participants had a mean age of 61.6 years. The sex ratio reflected a significant presence of women, constituting approximately two-thirds of participants.

Regarding smoking history, smokers and former smokers ranged from two to 108 pack-years, and eight participants had a smoking history of more than 20 pack-years.

From a histological point of view, the study cohort presented a diversity of tumor types, with adenocarcinoma and squamous cell carcinoma as the predominant forms. In addition, it included carcinoid tumors, neuroendocrine tumors, and a solitary metastatic tumor of gastrointestinal origin, diversifying the spectrum of malignant neoplasms considered.

As for tumor staging, based on the first altered computed tomography, 11 patients were identified in stage IA, six in stage IB, one in stage IIA, one in stage IIB, and one in stage IIIA. However, it is important to emphasize that this staging has limitations due to the absence of complementary tests.

When exploring the final tumor staging, the sample in question revealed a distribution containing seven patients categorized as stage IA, four as IB, four as IIB, and five as IIIA, highlighting varied clinical presentations. Regarding the technique chosen for intervention, there was a significant predominance of lobectomies (17) compared with pneumonectomy (two) and segmentectomy (one).

When comparing the initial and final stagings, we observed that 14 patients (70%) had progression in tumor staging, and among these, five (25%) developed lymph node dissemination.

Statistical tests did not reveal a significant association between tumor stages and qualitative characteristics, such as a history of smoking for more than 20 pack-years. 

Although the surgical treatment was performed at Hospital São Paulo, the patients were referred to the service through the Health Services Offer Regulation Center (CROSS) of the São Paulo Health Department, originating from Basic Health Units and public Emergency Departments. Access to the specialist also occurred through referral from other specialties within Hospital São Paulo and, exceptionally, some patients sought care autonomously through the institution Emergency Room after starting the investigation of the disease in the private sector.

### Specific results

During the data collection process, we identified the dates corresponding to the first abnormal chest CT scan, the first consultation with the thoracic surgeon, and the surgical procedures performed. These data, whose compilation is detailed in Table 2, served as basis for establishing the time intervals described in the Methods section.

**Table 2:** Interval in days between the stages of the study.

Interval	Mean (SD)	Median (range)
Diagnosis-Consultation	282 ± 383.2	113 (7-1527)
Consultation-Treatment	143 ± 133.5	73.5 (29-485)
Diagnosis-Treatment	425.6 ± 393.4	266 (90-1638)

Within the scope of this study, it is necessary to detail the interpretation of the Diagnosis-Treatment interval, which represents the combination of the Diagnosis-Consultation and Consultation-Treatment ones. The analysis of these data revealed that the total interval (Diagnosis-Treatment) ranged from 90 to 1,638 days. Statistical analysis reveals a median interval of 266 days and a mean of 425.6 days. An important observation emerges when comparing the mean and median values of the Diagnosis-Consultation interval with those obtained for the Consultation-Treatment interval, revealing that the time spent waiting for an appointment with the thoracic surgeon exceeded the time between the consultation and surgery.

Regarding diagnostic imaging, all patients who arrived at the service with imaging exams from outside the São Paulo Hospital underwent a new chest CT scan for comprehensive follow-up and updating of the clinical picture. In contrast, patients originating from the hospital itself and referring from other specialties were exempted from a new imaging test.

We identified no statistically significant associations between the Diagnosis-Consultation and Consultation-Treatment intervals, nor between the total interval (Diagnosis-Treatment) and the disease stages.

[Fig ch1]

**Graph 1 ch1:**
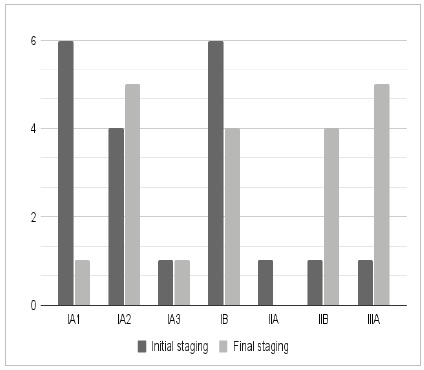
Distribution of the initial and final stagings.

[Fig ch2]

**Graph 2 ch2:**
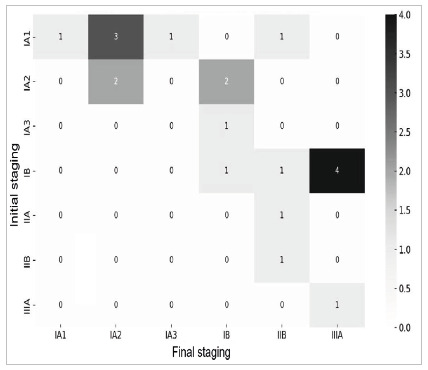
Distribution and advancement of the initial and final stagings.

## DISCUSSION

The median of 266 days identified for the interval between the first altered imaging test and the subsequent treatment appears as significant data, evidencing an extraordinarily prolonged duration. In addition, the staging progression of 70% of patients in this study highlights the impact that waiting for treatment can have on the final therapeutic outcome. However, many patients with lung cancer who had disease progression were not included in this series, either because the disease was already at an advanced stage or because they died during the waiting period. This represents much greater harm than that presented in our study. 

Notably, there is a divergence of the ranges found in this research compared with studies by other authors who explored similar variables. A study conducted at a referral hospital for cancer treatment in Japan reported a median of 870 days, as clinical follow-up was chosen for patients with ground-glass opacity-like alterations identified on computed tomography, which resulted in a longer interval for definitive treatment to be instituted^9^. Considering that lesions of this type may leave doubt as to their nature, as both benign and malignant conditions can have this presentation^10^, it is essential that these findings be followed up and in the suspicion of neoplasia and the investigation is complemented so that the diagnosis and intervention are not carried out late. In addition, because the time for tumor duplication with this tomographic pattern of lesion is slower, the follow-up of the lesion is a viable alternative until the oncological diagnosis is established, and interventions sparing lung parenchyma, such as segmentectomy, can be considered with the appropriate selection of patients^11^.

Other studies on the same topic, conducted in the United States of America (USA) and Canada, showed median intervals between the change in the imaging test and the start of treatment ranging from 36.5 to 105 days1^2^ ^-^ ^20^. In comparison with the findings of this study, all other authors revealed intervals shorter than the one found, of 266 days. This discrepancy reinforces the perception that the waiting time for treatment at a public health hospital in São Paulo, Brazil, where the data collection was conducted, is excessively prolonged, especially when compared with international data from developed countries.

Among the surveys, the authors who distinguished the intervals obtained according to the type of chosen treatment revealed medians greater than 80 days for the group submitted to surgery^12^ ^-^ ^17^. An important finding was a shorter wait for treatment of patients with stages III and IV^12^ ^-^ ^15^, part of which may be related to the symptomatic manifestation, which is generally absent in the group of patients with localized disease, and to the fewer complementary tests required prior to the establishment of the therapy of choice, since it did not involve surgical intervention. For this study, we could not perform this analysis because we included only patients undergoing surgical treatment, excluding the ones with advanced disease.

Only one of these studies had an interval of less than 50 days, reaching a median of 36.5 days, highlighting that 16% of the patients who were included experienced a waiting interval for treatment greater than 90 days^19^. In addition, the intervals were not established based on the type of treatment performed, so that patients undergoing chemotherapy, radiotherapy, and palliative care were included for analysis with patients undergoing surgery, and only 33% of individuals had local disease, with stages I and II, which may have contributed to the small interval reported.

In practice, a prolonged period until treatment is related to a lower probability of achieving disease-free survival^9^, impairing curative interventions. In agreement with this finding, another study showed that patients with stage III can be downgraded from curative treatment to palliative care in up to 29% of cases due to tumor progression in a period of 30 days^21^.

Similarly, the delay in treatment start also impacts patients diagnosed with lung cancer who have the disease in its initial stage. Other authors have reported that waiting periods of more than 12 weeks between the last imaging test and surgery resulted in lower survival and greater vulnerability to disease recurrence^22^ ^,^ ^23^.

An American study that evaluated these same variables in the public and private health sectors found that in the USA, patients admitted for lung cancer treatment in the public sector waited for almost twice the period for treatment compared with patients in the private sector, with medians of 76 and 45 days, respectively^20^. In addition to this difference in treatment waiting time, they identified that most patients in the public sector displayed more advanced stages, implying worse oncological outcomes^20^. Considering that in Brazil, the public and private health sectors show great disparity and access to health is marked by inequalities, it is estimated that significant differences such as those reported in the North American study may also be present among Brazilian health services.

When acknowledging that the longest interval was between the diagnosis and the consultation with the specialist, with 113 days of median waiting, it is important to highlight that there are factors related to the patient, the delay in referring the patient through the CROSS system, and the scarcity of specialists, which may have influenced this finding. According to the Medical Demography published by the Brazilian Medical Association, in 2023, there were about 1,268 specialists in thoracic surgery in Brazil, a specialty responsible for the surgical treatment of patients with lung neoplasms, which are concentrated in large capitals^24^. If such a delay was recorded in one of the largest Brazilian cities, concentrating many high-complexity hospital centers and a greater number of medical professionals and specialists, it is estimated that other locations may present even longer intervals.

Regarding the interval between the consultation with the specialist and surgery, which presented a median of 73.5 days, the main factors that may be responsible for this delay consist of waiting for the performance of tests, waiting for the biopsy, waiting for the results of the tests and biopsy, waiting list for surgery, shortage of operating rooms, among other factors, such as the postponement of surgeries or the loss of the exam date by the patient.

During the period of waiting for treatment, patients go through a spectrum of psychological anguish marked by elevated levels of worry and anxiety, which intensify proportionally as this interval is extended^25^. However, the impact of these prolonged intervals goes beyond mental well-being, as it represents a critical time window in which tumor growth and disease progression can occur^6^ ^-^ ^8^. This condition raises significant concerns about missing the opportunity for a curative surgical intervention. Consequently, this scenario may require other forms of treatment, considering that surgery as a definitive treatment may cease to be curative or no longer indicated as the disease progresses.

Recognizing that surgery is the most effective treatment, especially because it provides the best results in terms of curative therapy when the disease is in its early stages^3^, any progression observed during the waiting period for the start of therapy represents a detriment to the patient’s general prognosis, culminating in suboptimal results, as corroborated by several studies^9^ ^,^ ^22^ ^,^ ^23^. In addition to the impact on the health of each patient waiting for treatment in the public health network, this trajectory of disease progression imposes significant pressure on the public system, as advanced stages require higher-cost therapies. This financial burden can be attributed to the inflated costs of systemic treatment compared with surgical treatment, encompassing prolonged periods of chemotherapy and radiotherapy that can extend for more than six months, inevitably exceeding the costs of surgical interventions^26^ ^,^ ^27^.

According to a study conducted at the Brazilian National Cancer Institute in 2015, patients with advanced-stage lung cancer had an average cost of treatment of R$ 8,929.82, while the supplementary health sector displayed an average cost of R$ 52,649.01 for first-line treatment^27^ ^,^ ^28^. Both studies identified that a significant part of these costs was allocated to the financing of the chemotherapy and radiotherapy used. On the other hand, the amount transferred by the Unified Health System (SUS) to hospitals for a pulmonary lobectomy, the gold standard for resection of lung cancer, is R$ 3,282.83, while a chemotherapy session is funded by R$1,100.00, as informed by the SUS Procedure Table Management System^29^. Although SUS does not pay 100% of the value of the procedures performed, the disparity between treatments is exorbitant and represents how much the public system is burdened by these therapies.

In view of the significant impact of geographic disparities, it is evident that populations living in areas characterized by a high concentration of cancer treatment centers benefit from easy access to therapeutic interventions^30^. This observation suggests that, in regions less privileged than the city of São Paulo or among marginalized populations in the city itself, the intervals between diagnosis and treatment can potentially exceed those identified in the present study. This possibility highlights a contradiction with the fundamental principles that sustain SUS, which emphasize the provision of health services with equity and universality, without favoring specific groups or populations^31^. Consequently, this discrepancy observed in access to medical interventions based on geographic and socioeconomic factors determines an urgent need for systemic improvements to ensure that the principles of SUS are complied with and that better results in cancer treatment are achieved.

Based on the findings of our study and the research referenced above, it is evident that faster access to the health system, to the general practitioner, and to the specialist physician benefits patients. The problem in which Brazil finds itself today consists of poor management of resources, so that in addition to the shortage of medical professionals, these are concentrated in large urban centers, where the infrastructure may still be precarious, but has better conditions than in other locations^32^. Therefore, to facilitate access to the health system, it is essential that there is investment in an initial process of decentralization of these professionals and in measures to solve the shortage of doctors.

Another key factor that can contribute to the reduction of this prolonged interval is the improvement of the public health system infrastructure, favoring the performance of tests such as chest computed tomography, which is essential for the initial investigation of lung cancer. As a result, strategies such as lung cancer screening may be employed. Brazilian medical institutions are in favor of instituting it, given the benefits of screening in selected patients, such as the identification of lesions in initial stages, ensuring better health outcomes^33^. 

The limitations of this study are its retrospective design, which had access to the hospital database for data collection. This may imply the loss of useful information for the research and the limited number of participants. A factor we did not explore is the acquisition of similar data in the private health network to compare the interval faced by patients in these two systems. Other dimensions could also have been addressed, such as the psychological issue of patients in this waiting period and the patients’ perception of the interval in which they waited for treatment.

## CONCLUSION

The interval faced by patients surgically treated for lung cancer in the public health system of the city of São Paulo was extremely large and higher than that reported by other authors in developed countries. This long waiting can compromise treatment oncological outcomes, resulting in lower survival and higher recurrence rate, in addition to affecting the quality of life causing psychological damage to those who wait for so long for surgery in this condition. Given the socioeconomic and regional inequalities in the country, it is estimated that these results will not be repeated in the private sector and in other locations. To ensure better care for cancer patients, it is essential that investments are made to reduce these intervals.
